# Distribution and Outcomes of a Phenotype-Based Approach to Guide COPD Management: Results from the CHAIN Cohort

**DOI:** 10.1371/journal.pone.0160770

**Published:** 2016-09-29

**Authors:** Borja G. Cosio, Joan B. Soriano, Jose Luis López-Campos, Myriam Calle, Juan José Soler, Juan Pablo de-Torres, Jose Maria Marín, Cristina Martínez, Pilar de Lucas, Isabel Mir, Germán Peces-Barba, Nuria Feu-Collado, Ingrid Solanes, Inmaculada Alfageme, CHAIN study

**Affiliations:** 1 Department of Respiratory Medicine, Hospital Son Espases-IdISPa, Palma de Mallorca, Spain; 2 CIBER de Enfermedades Respiratorias (CIBERES), Instituto de Salud Carlos III, Madrid, Spain; 3 Instituto de Investigación Hospital Universitario de la Princesa (IISP), Universidad Autónoma de Madrid, Cátedra UAM-Linde, Madrid, Spain; 4 Department of Respiratory Medicine, Hospital Universitario Virgen del Rocío- IBiS, Sevilla, Spain; 5 Department of Respiratory Medicine, Hospital Clinico San Carlos, Madrid, Spain; 6 Department of Respiratory Medicine, Hospital Arnau de Vilanova, Valencia, Spain; 7 Department of Respiratory Medicine, Clínica Universidad de Navarra, Pamplona, Spain; 8 Department of Respiratory Medicine, Hospital Universitario Miguel Servet, Zaragoza, Spain; 9 Department of Respiratory Medicine Hospital Central de Asturias, Oviedo, Spain; 10 Department of Respiratory Medicine, Hospital Gregorio Marañon, Madrid, Spain; 11 Department of Respiratory Medicine, Hospital Son Llátzer, Palma de Mallorca, Spain; 12 Department of Respiratory Medicine, Fundación Jimenez Diaz, Madrid, Spain; 13 Department of Respiratory Medicine, Hospital Universitario Reina Sofía, Cordoba-IMIBIC.UCO, Spain; 14 Department of Respiratory Medicine, Hospital San Pablo y la Santa Cruz, Barcelona, Spain; 15 Department of Respiratory Medicine, Hospital Universitario de Valme, Sevilla, Spain; 16 Department of Respiratory Medicine, Hospital Ntra. Sra. de Candelaria, Tenerife, Spain; Lee Kong Chian School of Medicine, SINGAPORE

## Abstract

**Rationale:**

The Spanish guideline for COPD (GesEPOC) recommends COPD treatment according to four clinical phenotypes: non-exacerbator phenotype with either chronic bronchitis or emphysema (NE), asthma-COPD overlap syndrome (ACOS), frequent exacerbator phenotype with emphysema (FEE) or frequent exacerbator phenotype with chronic bronchitis (FECB). However, little is known on the distribution and outcomes of the four suggested phenotypes.

**Objective:**

We aimed to determine the distribution of these COPD phenotypes, and their relation with one-year clinical outcomes.

**Methods:**

We followed a cohort of well-characterized patients with COPD up to one-year. Baseline characteristics, health status (CAT), BODE index, rate of exacerbations and mortality up to one year of follow-up were compared between the four phenotypes.

**Results:**

Overall, 831 stable COPD patients were evaluated. They were distributed as NE, 550 (66.2%); ACOS, 125 (15.0%); FEE, 38 (4.6%); and FECB, 99 (11.9%); additionally 19 (2.3%) COPD patients with frequent exacerbations did not fulfill the criteria for neither FEE nor FECB. At baseline, there were significant differences in symptoms, FEV_1_ and BODE index (all p<0.05). The FECB phenotype had the highest CAT score (17.1±8.2, p<0.05 compared to the other phenotypes). Frequent exacerbator groups (FEE and FECB) were receiving more pharmacological treatment at baseline, and also experienced more exacerbations the year after (all p<0.05) with no differences in one-year mortality. Most of NE (93%) and half of exacerbators were stable after one year.

**Conclusions:**

There is an uneven distribution of COPD phenotypes in stable COPD patients, with significant differences in demographics, patient-centered outcomes and health care resources use.

## Introduction

COPD is a heterogeneous disease characterized by chronic airflow obstruction in which exacerbations and comorbidities, among other clinical factors, can contribute to its severity [1]. In order to approach this heterogeneity, an attempt to group patients with similar characteristics that could be associated to a differential clinical outcome has been done by using the term clinical phenotype [2]. Clear examples of COPD phenotypes associated to different outcomes have been recently shown, such as the frequent exacerbator [3] or the overlap COPD and asthma phenotypes [4]. However, international guidelines, such as the Global Initiative in Obstructive Lung Diseases (GOLD), do not recognize these clinical characteristics when addressing the best therapeutic options to treat these patients, relying this decision on lung function, symptoms and history of exacerbations. However, the overwhelming evidence that supports different response to therapy in patients with specific characteristics (inhaled steroids in patients with eosinophilia [5], roflumilast in patients with chronic bronchitis [6], etc) raises the need for a more comprehensive approach to treat these patients.

The new Spanish Guideline for COPD (GesEPOC) [7] proposes a different algorithm for pharmacologic treatment based on four phenotypes: the non-exacerbator (NE) with either emphysema or chronic bronchitis phenotype; the COPD and Asthma overlap phenotype (ACOS); the frequent exacerbator with emphysema (FEE) and the frequent exacerbator with chronic bronchitis (FECB). Severity is assessed by the BODE or the BODEX indexes, and pharmacologic treatment is adjusted according to phenotype and severity. A stepwise approach in the pharmacologic treatment is proposed, with long-acting bronchodilators as the mainstay of treatment, but differentiating specific therapies for the different phenotypes adjusted for severity (inhaled steroids from early stages in ACOS, roflumilast for FECB, etc). This innovative approach has been adopted after the consensus of the different players in the health-care management of COPD patients, namely respiratory medicine specialists, primary care, and internal medicine—by their respective scientific societies- and supported by the health-care authorities. Miravitlles at al have recently shown that different phenotypes showed different demographic and clinical characteristics [8]. However, long-term stability of these phenotypes is largely unknown.

In order to explore the distribution of these four COPD phenotypes and their differences in terms of demographic data and one-year patient related outcomes, data from a Spanish multicenter prospective cohort with multidimensional evaluation of COPD patients (COPD History Assessment In SpaiN (CHAIN)) were analyzed.

## Methods

All patients signed an informed, written consent form, which was previously approved by each one of the Ethics Committee in every participating center. The study was approved by the Ethics Committee of the Balearic Islands.

Methodology and recruitment strategy has been published elsewhere [9]. Briefly, COPD was defined by smoking history ≥10 pack-years and a post-bronchodilator FEV_1_/FVC <0.7 after 400 μg of inhaled albuterol. The main goal of this prospective observational study is to perform a multidimensional evaluation of the evolution of COPD patients to better define its natural history and potential clinical phenotypes (ClinicalTrials.gov Identifier: NCT01122758). The recruitment period was between January 15, 2010 and March 31, 2012. Data analyzed in the present study come from the baseline and one year assessments. Demographic and clinical data were evaluated at baseline and at first annual visit: anthropometric data (age, gender and BMI), comorbidities (Charlson index), smoking history, dyspnea (mMRC scale), exacerbations in the previous year, health status by the validated Spanish version of the COPD Assessment Test (CAT) and Clinical COPD (CCQ) questionnaire, anxiety and depression (HAD questionnaire), pharmacologic treatments, respiratory function (arterial blood gases, spirometry, lung volume and diffusion capacity), exercise capacity by six-minute walk distance (6MWD) and BODE index. Data of the patients were anonymized in a database with a hierarchical access control in order to guarantee secure information access.

### Clinical and physiological measurements

Trained staff in a personal interview obtained the following information at the time of recruitment and at yearly appointments: age, gender and the body mass index (BMI), calculated as the weight in kilograms divided by height in meters. A specific questionnaire was used to determine smoking status (current or former) and smoking history (pack-years). Chronic bronchitis was defined by the presence of cough and sputum production during three consecutive months in two consecutive years. The presence or absence of emphysema was considered according to radiological (presence of emphysema in CT scan as reported by the local radiologist) and/or functional criteria (DLCO<80%). The presence of comorbidities was evaluated by the Charlson index.

Pulmonary function tests were performed following ATS guidelines [10]. The diffusion capacity for carbon monoxide (DLCO) was determined by the single breath technique following the ERS/ATS guidelines. The 6MWD test was measured as the better of two walks separated by at least 30 minutes. Dyspnea was evaluated by the modified Medical Research Council (mMRC) scale. The percentage of forced expiratory volume at first second (FEV_1_%), BMI, 6MWD and MMRC values were integrated into the BODE index as previously described [11]. Exacerbations were defined by use of antibiotics (mild exacerbations), steroids or both (moderate exacerbations) captured from a diary of exacerbations (handled between the patient, the primary care physician and the chest physician) or admission to hospital (severe exacerbations) related to worsening of respiratory symptoms.

### Definition of phenotype

The NE phenotype was considered if the patient had less than two exacerbations requiring antibiotics, oral steroids or hospitalization in the preceding 12 months, regardless of the presence of emphysema or criteria for chronic bronchitis. ACOS definition was based on previously published criteria [12]. Accordingly, ACOS was defined in the presence of one major criteria (namely, a previous history of asthma, or a bronchodilator response to albuterol higher than 15% or 400 ml) or two minor criteria (blood eosinophils>5%, immunoglobuline E>100 IU, or two separated bronchodilator responses to albuterol higher than 12% and 200 ml).

**Fig 1:**
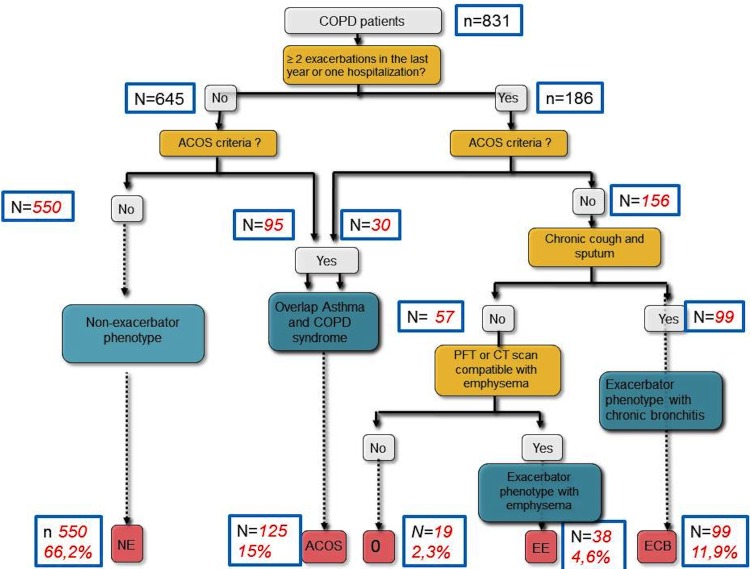
Flow-chart and algorithm of patients and phenotypes.

The frequent exacerbator phenotype was defined by the presence of, at least, two exacerbations requiring antibiotics, oral steroids or one hospitalization in the preceding 12 months and separated by at least four weeks. Frequent exacerbator were also divided according to the presence of chronic bronchitis (defined by the presence of cough and sputum production for at least 3 months during two consecutive years) regardless of the presence of emphysema (CB phenotype), the presence of emphysema in the absence of chronic bronchitis (FEE phenotype), or the presence of ACOS criteria. If patients with frequent exacerbations did not fit into any of the previously set phenotypes were designated as group 0. The distribution of the four different phenotypes according to these criteria were analyzed following a proposed algorithm (Fig 1).

### Patients and variables

COPD patients participating in the CHAIN cohort were included. Association between baseline phenotype and one-year clinical outcomes, mainly CAT, FEV_1_, mMRC score, BODE and exacerbations during the follow-up period were evaluated.

### Statistical analysis

Data are summarized as relative frequencies for categorical variables, mean (SD) for normally distributed scale variables and median (5th‒95th percentile) for ordinal or non-normal scale variables. Comparisons were performed using Pearson’s Chi-square for categorical variables and ANOVA for continuous variables, according to the variables type and distribution. Standard Kaplan-Meier statistics were applied, including a Cox model to determine the significance of survival probabilities by covariates (age, gender and GOLD severity) of COPD patients according to different phenotypes. Significance level was established as a two-tailed p value <0.05. Analyses were performed using SPSS statistic package version 20.0 Inc. (Chicago, IL, USA).

## Results

### Distribution of phenotypes

Eight hundred and thirty-one patients included in the CHAIN cohort were classified in different phenotypes according to a proposed algorithm (Fig 1). Most patients were classified as non-exacerbator phenotype (66.2%), 15% fulfilled criteria for ACOS, and the remaining 18.8% were classified as exacerbator phenotype. Of these, 63.5% of patients were FECB phenotype, 24.3% were FEE phenotype and 12.2% (19 patients, 2.3% of the entire COPD population) were frequent exacerbators that did not fulfill criteria for either chronic bronchitis or emphysema (group 0).

**Fig 2:**
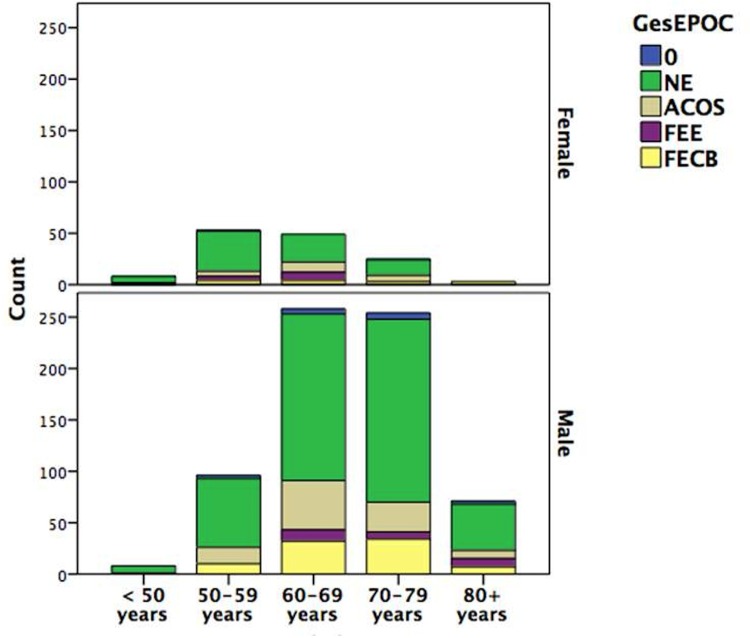
Distribution of phenotypes by age and gender.

Distribution of phenotypes by age and gender showed a marked increase in the exacerbator-type phenotype (FEE, FECB or 0) with age (p<0.05), especially in women (Fig 2).

### Characteristics of patients according to phenotype

Baseline socio-demographic and clinical characteristics are shown in Table 1.

**Table 1:** Demographic and clinical characteristics, by phenotype.

	0	NE	ACOS	FEE	FECB	p
	19 (2.3%)	550 (66.2%)	125 (15.0%)	38 (4.6%)	99 (11.9%)	
**Female, n (%)**	2 (10.5)	90 (16.4)	23 (18.4)	12 (31.6)	14 (14.1)	0.126
**Age, mean ± SD**	69.0±9.4	67.4±9.1	66.5±8.7	68.4±8.7	69.5±8.1	0.113
**Pack-year, mean ± DE**	50.8±28.4	56.3±28.7	53.2±26.2	52.9±26.3	60.8±30.0	0.270
**Current smoker, n (%)**	2 (10.5)	156 (28.4)	44 (35.2)	10(26.3)	28 (28.3)	0.218
**BMI, mean ± SD**	28.7±4.8	28.0±5.7	29.0±5.5	28.0±4.8	27.8±4.8	0.367
**Symptoms, n (%)**						
**-cough and sputum**	0 (0.0)	315 (57.3)	75 (60.0)	0 (0.0)	99 (100.0)	<0.001
**-dyspnea (mMRC >2)**	10 (52.6)	233 (42.4)	56 (44.8)	16 (43.2)	67 (67.7)	<0.001
**CAT (m ± SD)**	11.28±7	11.74±7	12.02±7,5	11.61±6,1	17.14±8,2	<0.001
**FEV**_**1**_**%, m ± SD**	58.2±19.8	60.7±21.1	61.2±18.1	55.3±15.7	52.9±19.4	0.004
**FVC%, m ± SD**	83.3±21.4	86.7±23.3	84.9±18.5	86.3±24.7	80.1±23.1	0.116
**FEV**_**1**_**/FVC, m ± SD**	53.1±12.6	52.9±11.5	54.8±10.9	49.1±9.9	49.2±10.9	0.001
**Prevalence of GOLD airflow limitation, n (%)**						0.074
**GOLD I**	2 (10.5)	104 (19.0)	21 (16.8)	3 (7.9)	11 (11.0)	
**GOLD II**	11 (57.9)	263 (48.0)	69 (55.2)	21 (55.3)	39 (39.4)	
**GOLD III**	4 (21.1)	120 (21.9)	25 (20.0)	10 (26.3)	29 (29.3)	
**GOLD IV**	2 (10.5)	61 (11.1)	10 (8.0)	4 (10.5)	20 (20.2)	
**BODE, mean ± SD**	1.8±1.6	1.9±1.8	1.9±1.8	2.0±1.7	3.0±2.5	<0.001
**BODE quartile, n (%)**						0.001
**0–2**	12 (70.6)	357 (68.8)	78 (65.5)	26 (70.3)	45 (47.9)	
**3–4**	3 (17.6)	114 (22.0)	33 (27.7)	9 (24.3)	26 (27.7)	
**5–6**	2 (11.8)	39 (7.5)	5 (4.2)	1 (2.7)	16 (17.0)	
**7–10**	0 (0.0)	9 (1.7)	3 (2.5)	1 (2.7)	7 (7.4)	
**DLCO <80%, n (%)**	0 (0.0)	242 (44.0)	50 (40.0)	25 (65.8)	47 (47.5)	<0.001
**Emphysema by CT scan, n/N (%)**	0/2 (0%)	125/197 (63.5%)	19/35 (54.3%)	17/19 (89.5%)	25/34 (73.5%)	0.019
**Moderate-severe exacerbation in previous year, n (%)**	9 (47.4)	39 (7.1)	22 (17.6)	25 (65.8)	73 (76.7)	<0.001

Patients in different phenotypes were predominantly male and showed similar age, smoking history, BMI, or spirometric severity (p>0.05). There were statistically significant differences in symptoms, CAT score, FEV1, FEV1/FVC and BODE index between groups, being the FECB phenotype the most symptomatic with higher BODE score. By definition, and as a consequence of algorithm application, exacerbations or the presence of emphysema were different among groups. Patients with frequent exacerbations without criteria for chronic bronchitis or emphysema (group 0) were not different in clinical characteristics other than cough and sputum production or the presence of emphysema that were generated by the application of the algorithm. Comorbidities, measured by the Charlson index, were similar among all groups. Only previous diagnosis of asthma, as a sole criterion to define ACOS, was different with the other phenotypes. Anxiety and depression were also present in similar proportion among the different phenotypes. Again, patients in group 0 did not show a differential pattern of comorbidities (Table 2).

**Table 2:** Distribution of selected comorbidities, by phenotype.

	0	NE	ACOS	FEE	FECB	p
	19 (2.3%)	550 (66.2%)	125 (15.0%)	38 (4.6%)	99 (11.9%)	
**Charlson, mean ± SD**	1.6±1.7	1.2±1.5	1.2±1.6	1.2±1.5	1.6±1.7	0.361
**Asthma, n (%)**	0	0	28 (22.4)	0	0	<0.001
**Dislypemia, n (%)**	7 (36.8)	172 (31.3)	46 (36.8)	17 (44.7)	33 (33.3)	0.399
**Diabetes, n (%)**	3 (15.8)	100 (18.2)	25 (20.0)	5 (13.2)	19 (19.2)	0.902
**Cardiopatía, n (%)**	3 (15.8)	86 (15.6)	14 (11.2)	5 (13.2)	18 (18.2)	0.654
**OSA, n (%)**	2 (10.5)	59 (10.7)	15 (12.0)	2 (5.3)	9 (9.1)	0.800
**Anxiety, mean ± SD**	10.1±5.4	11.1±5.0	10.7±4.8	11.1±5.0	12.0±4.4	0.343
**Anxiety, n (%)**						0.285
**- no**	5 (33.3)	121 (26.2)	27 (24.8)	8 (24.2)	14 (15.6)	
**-potential**	3 (20.0)	84 (18.2)	28 (25.7)	7 (21.2)	16 (17.8)	
**-confirmed**	7 (46.7)	257 (55.6)	54 (49.5)	18 (54.5)	60 (66.7)	
**Depression, mean ± SD**	7.9±5.4	8.6±4.6	8.3±4.9	8.5±4.9	9.7±5.3	0.313
**Depression, n (%)**						0.650
**-no**	7 (46.7)	204 (48.1)	49 (48.0)	14 (50.0)	33 (38.4)	
**-potential**	4 (26.7)	68 (16.0)	17 (16.7)	3 (10.7)	13 (15.1)	
**-confirmed**	4 (26.7)	152 (35.8)	36 (35.3)	11 (39.3)	40 (46.5)	

Pharmacological treatments for COPD were statistically different among the different phenotypes, being the exacerbator phenotypes (FEE and FECB) those with the highest use of respiratory drugs (Table 3), while those in group 0 had the highest anticholinergics and theophylline use.

**Table 3:** Distribution of respiratory medication use, by phenotype.

	0	NE	ACOS	FEE	FECB	p
	19 (2.3%)	550 (66.2%)	125 (15.0%)	38 (4.6%)	99 (11.9%)	
**Anticholinergics, n (%)**	18 (94.7)	398 (72.5)	82 (65.6)	33 (86.8)	88 (88.9)	<0.001
**Beta2-agonists, n (%)**	15 (78.9)	396 (72.0)	91 (72.8)	32 (84.2)	85 (85.9)	0.029
**Inhaled steroids, n (%)**	14 (73.7)	344 (62.5)	79 (63.2)	29 (76.3)	78 (78.8)	0.009
**Theophylline, n (%)**	6 (31.6)	43 (7.8)	6 (4.8)	4 (10.5)	18 (18.2)	<0.001

### Longitudinal assessment

Four hundred and ninety-four patients were re-studied after one year, 333 patients from the non-exacerbator phenotype, 77 from ACOS and 84 from the exacerbator phenotypes. There were 270 drop-outs and 67 deaths during follow-up.

**Fig 3:**
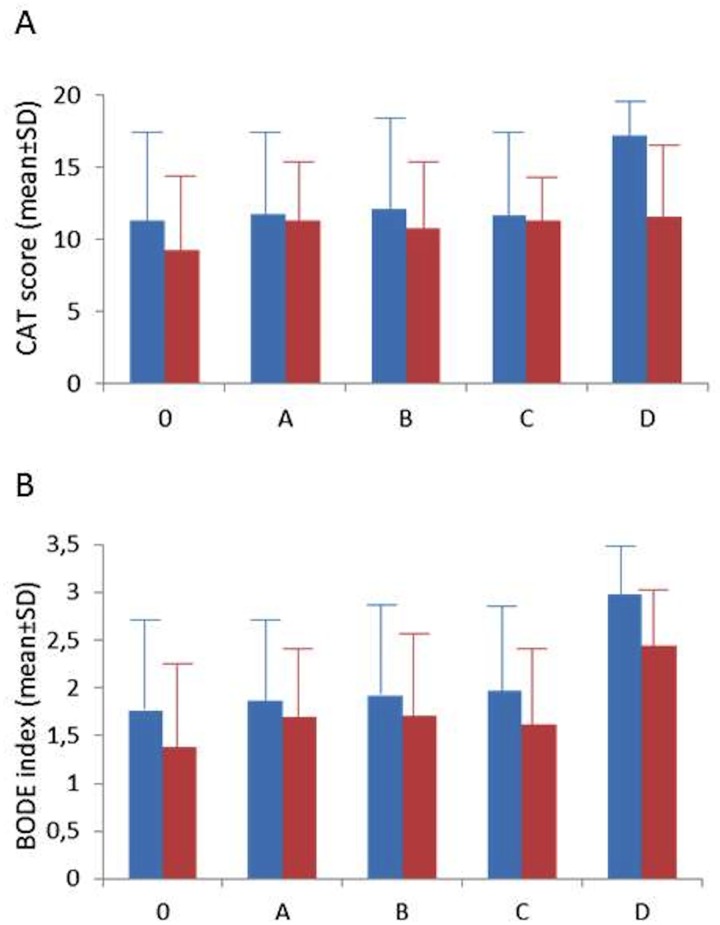
Baseline and one-year scores for CAT (panel A) and BODE (panel B).

After one-year of follow-up patients showed statistically significant differences in CAT-score (p<0.05) and non-significant differences in BODE index (Fig 3) compared with their baseline characteristics. Smoking cessation was observed in 12,6% of the population.

**Fig 4:**
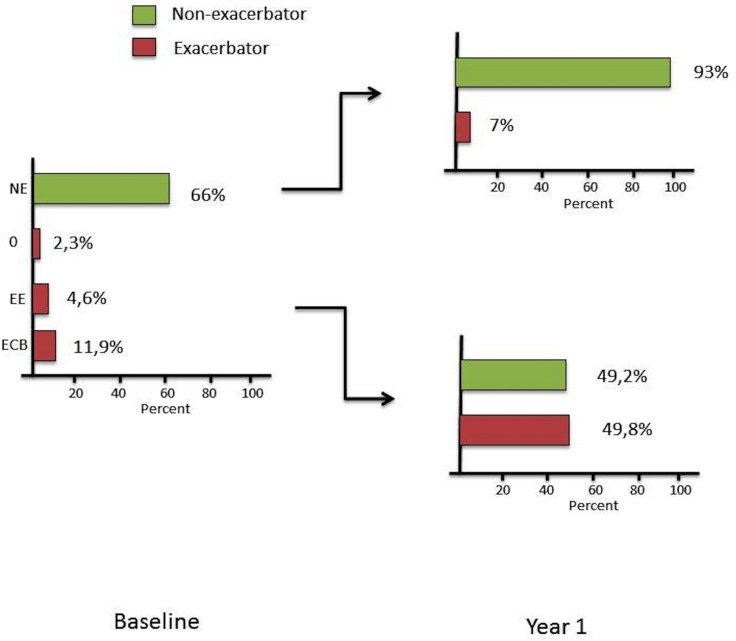
Baseline and one-year distribution of phenotypes.

Moderate-severe exacerbation rates remained also significantly different among phenotypes, with a higher proportion of patients with moderate-severe exacerbations among the FEE and FECB phenotypes. After one-year of follow-up, 97% of NE phenotype and nearly half (49.8%) of patients with frequent exacerbations (FEE, FECB or 0) remained in the same phenotype (Fig 4).

**Fig 5:**
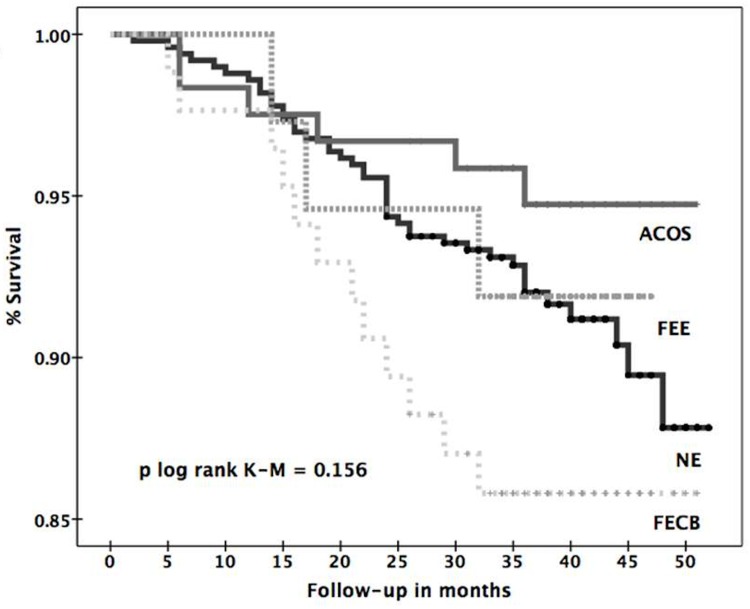
Kapplan-Mayer survival curve by phenotype.

Finally, sixty-seven deaths were observed during one-year of follow-up, with no differences between phenotypes (p log rank Kaplan Mayer 0.156, Fig 5). However, note that the comparison of one-year mortality of the extreme groups FECB vs ACOS was significant with a p = 0.026, but not so within the other two. The causes of death were respiratory failure (15 patients), lung cancer (12), other cancer (7), cardiovascular disease (7), pneumonia (3), COPD exacerbation (1), other causes (5) and unknown (17) (data not shown).

## Discussion

We have reported the clinical characteristics and distribution of previously proposed clinical phenotypes in a large cohort of COPD patients followed-up for one year. The GesEPOC proposed approach separates patients by severity of airway obstruction and BODE, symptoms, the presence of exacerbations, and emphysema, and by pharmacological treatment. However, a small proportion of patients (2.3%) remained unclassified by this algorithm. Most patients classified according to these clinical phenotypes remained in the same category after one year of follow-up. Moreover, the FECB phenotype was the most symptomatic with higher BODE score and showed a trend to worse survival after one year.

### Interpretation of novel findings

Clinical phenotypes in COPD have been defined as a single or combination of disease attributes that describe differences between individuals with COPD as they relate to clinically meaningful outcomes (symptoms, exacerbations, response to therapy, rate of disease progression or death) [2]. Our results demonstrate that the proposed GesEPOC phenotypes are associated to meaningful outcomes such as CAT, BODE, lung function, or exacerbations. Although patients in our cohort were not treated according to a phenotype-based strategy, they show differences in pharmacological treatments among the different phenotypes, which reflect the application of personalized medicine in daily clinical practice, even at a time when lung function-based guidelines were used. Interestingly, ACOS did not have the highest proportion of patients on inhaled corticosteroids, since it was not specifically recognized by the current guidelines at the time of recruitment.

Nevertheless, the proposed algorithm leaves a number of patients (2.3%) without a clear phenotype other than frequent exacerbator, without chronic bronchitis or emphysema. This also happens when trying to apply the Spanish GesEPOC phenotype-based guideline in a daily clinical basis and probably is reflecting a pool of patients in whom exacerbations are triggered by other comorbid conditions, since they show the highest Charlson score (Table 2), similar to FECB patients.

### Previous studies

Other groups have previously shown that different COPD phenotypes are related to clinically meaningful outcomes. Soler-Cataluña et al. demonstrated that patients with frequent exacerbations have worse survival than non-exacerbator patients [13]. This was further explored by Hurst et al. in the ECLIPSE cohort [3] demonstrating that exacerbations became more frequent (and more severe) as the severity of COPD increased and that the single best predictor of exacerbations, across all GOLD stages, was a history of exacerbations. Our results are in keeping with those previous observations.

ACOS is now recognized as a distinct COPD clinical phenotype, and deserves special attention in international guidelines for both diseases, namely GOLD and GINA [14]. We have previously shown that the clinical criteria used to define ACOS in the present study are useful and easy to apply in this cohort [12]. Prevalence of ACOS found here (15%) is similar to previously described in other cohorts such as COPDGene (13%)[4]. Differences in clinical outcomes in this specific phenotype are discussed elsewhere [12].

Chronic bronchitis has also been associated with worse respiratory symptoms and higher risk of exacerbations in well-characterized cohorts of COPD patients such as COPDGene [15] or PLATINO [16].

Emphysema is associated to mortality in COPD patients [17]. Different cluster analysis studies have identified a cluster of patients at very low risk of mortality, who had mild respiratory disease and low rates of comorbidities and a different cluster with emphysema and higher risk of mortality [18,19]. Rennard et al demonstrated that a cluster of patients with severe emphysema, low FEV1 and the highest exacerbation and COPD hospitalization rate was present in the ECLIPSE cohort [20]. We have shown here that these phenotypic characteristics are stable when evaluating them after one year.

The distribution of the four clinical phenotypes proposed here has been recently described in a cross-sectional study [8] with similar proportion to our population: 60.6% non-exacerbators, 15.9% ACOS patients, 19.3% exacerbators with chronic bronchitis and 4.3% exacerbators without chronic bronchitis. In this study, different phenotypes showed different demographic and clinical characteristics as well as impact on health-related quality of life and mood.

### Clinical implications

The recognition of a predominant phenotype may help to guide the best therapeutic option for a single patient, and this is the rationale for the novel approach of phenotype-oriented guidelines [7,21,22]. However, the proposals are based in expert consensus based on literature review and clinical expertise, since no evidence exists proving that a phenotype approach is better than the currently recommended approach based on lung function, symptoms and exacerbations [1]. Our study demonstrates that a phenotype-based classification is easy to apply in a majority of COPD patients, and shows the distribution and clinical characteristics of patients classified by a clinical algorithm. Those patients with frequent exacerbations and no clear emphysema or chronic bronchitis are likely to be related with comorbidities, as other cohorts have previously identified in cluster analysis [18].

The approach based on phenotypes could be different to the current international recommendations when we look at survival. As a sentivity analysis, we compared the survival curves of the four GesEPOC proposed phenotypes (Fig 5), with the latest GOLD 2013 staging, that includes CAT, mMRC and CCQ to assess the symptoms domain (data not shown). The GOLD 2013 staging had a better prognostication for 1-year mortality (log-rank test = 0.004) versus the GesEPOC staging (log-rank test = 0.156), but with a greater mortality in B than C stages, which severely limits its usability, as highlighted elsewhere [23].

### Limitations

Our study has several limitations. Firstly, although patients from this well-characterized cohort are classified by a clinical algorithm into different phenotypes, patients may share characteristics of different phenotypes at the same time, which could lead to a different staging along the natural history of the patient´s disease. However, phenotype characteristics remained reasonably stable, at least, after one-year of follow-up. Moreover, to address the factors that shift patients from one to other phenotype would be of interest but it was beyond the scope of our study. Secondly, there is a loss of patients during follow-up that could bias the interpretation of outcomes. This fact is also affecting the analysis of survival, and, added to the low number of deaths observed, could justify the lack of differences in the analysis of mortality. And thirdly, our patients are treated according to the current guidelines by the time of recruitment, but most likely also according to physician judgment, which could imply a kind of “phenotyping” patients already.

## Conclusions

We have shown that there is an uneven distribution of COPD phenotypes in stable COPD patients, with significant demographic, clinical, and use of health resources differences. The differences observed remain stable after one-year of follow up. The phenotype approach helps the clinician to identify the patients that can benefit more of a specific treatment. Further studies are needed to demonstrate whether this approach is associated with better clinical outcomes than the current approach based on lung function, symptoms and exacerbations.
