# Correction: Distribution and Outcomes of a Phenotype-Based Approach to Guide COPD Management: Results from the CHAIN Cohort

**DOI:** 10.1371/journal.pone.0166257

**Published:** 2016-11-02

**Authors:** The PLOS ONE Staff

Dr. Ciro Casanova is not included in the author byline. He should be listed as the last author as Ciro Casanova on behalf of the CHAIN study. He is affiliated with the Department of Respiratory Medicine, Hospital Ntra. Sra. de Candelaria, Tenerife, Spain. The contributions of this author are as follows: Recruited patients and reviewed the manuscript.

The publisher apologizes for the error.
